# Mass safe male circumcision: early lessons from a Ugandan urban site - a case study

**Published:** 2012-12-28

**Authors:** Moses Galukande, Denis Bbaale Sekavuga, Kevin Duffy, Nicholas Wooding, Sam Rackara, Florence Nakaggwa, Teddy Nagaddya, Alex Emmanuel Elobu, Alex Coutinho

**Affiliations:** 1 International Hospital Kampala, Uganda; 2 Infectious Diseases Institute, Makerere University, Kampala, Uganda; 3 International Medical Group; 4 International Health Sciences University, Kampala, Uganda; 5 Department of Surgery, Mulago Hospital, Kampala, Uganda

**Keywords:** Safe male circumcision, public and private partnership, task shifting

## Abstract

**Introduction:**

It has been proven in several randomized clinical trials that HIV transmission from female to male is reduced by 60% and more among circumcised males. The national target for Uganda by 2015 is to circumcise 4.2 million adult males, an unprecedented number requiring a pragmatic approach and effective model(s) to deliver this target. The objective of the study was to describe early lessons learnt at a start up of a mass safe male circumcision (SMC) program in an urban Ugandan site, implemented through task shifting and a private public partnership approach.

**Methods:**

A case study of an urban SMC site in Uganda's capital, Kampala with a catchment population of approximately 0.8 million adult males aged between 15 and 49 years. Client enrollment was voluntary; mobilization was by word of mouth and through the media. Non Physician clinicians (NPC) carried out the majority of the SMCs. The SMC and voluntary counseling and testing (VCT), adverse events (AE) management and follow up were done as per set national guidelines. The supervision was by a public and private service provider. All clients were consented.

**Results:**

A total of 3000 males were circumcised in 27 days spread over four months. The AE rate was 2.1% all AEs were mild and reversible. No deaths occurred. The work rate was 111 SMCs per day. There was sufficient demand for SMC despite minimal mobilization effort. The bulk of the SMC work was successfully carried out by the NPCs.

**Conclusion:**

Private Public Partnership and task shifting approaches were successful at the start up phase and we anticipate will be feasible for the scale up.

## Introduction

It has been proven in several randomized clinical trails (RCTs) that HIV transmission from female to male is reduced up to 60% among circumcised males [1–3]. Some of the main reasons suggested are, HIV target cells (Langerhans cells which express HIV-1 receptor) in the foreskin are markedly reduced by circumcision [4–6] as well as the resultant keratinization of the glans and residual mucosal "cuff" [5], the prepuce may be susceptible to mild trauma during intercourse facilitating infection whereas the moist and warm environment under the prepuce harbor other organisms that cause sexually transmitted infection (STI) and is conducive for prolonged viral survival [6, 7].

It has been observed in ecological studies that in populations where the male circumcision prevalence is over 80%, the HIV prevalence is less than 5% and where the male circumcision prevalence is low the HIV prevalence is high. The average Uganda male circumcision prevalence is 25%. The national target for Uganda over the next 5 years is 4.2 million circumcisions (requiring at least 840,000 per year). This is unprecedented and requires a large concerted effort and requires multiple implementers since government alone doesn't have the capacity to implement. The over-arching goal is to reduce the prevalence of infection by 25% in 20 years translating into saving several billions of US dollars [8–10]. This paper describes success factors and challenges of a mass SMC start up at a Ugandan urban site.

### Study context

A service contract between two provider institutions one private (International Hospital Kampala) and one public service provider (Infectious Diseases Institute) was made. The private institution (IHK) was to fulfill provision of a minimum safe male circumcision [11] package including: STI screening and treatment, condom distribution, VCT and male circumcision. Only adult males (above 15 years) were to be circumcised under local anesthesia. All children below the age of 15 years were to be excluded. The terms of reference were discussed and agreed upon prior to commencement of the exercise.

The exercise was executed by trained team of health professionals. The team was initially composed of two specialist surgeons (as instructors), three medical officers (supervisors), eight graduate nurses, six clinical officers, twenty nurses (mostly registered nurses), three cleaners/porters, four certified counselors and two data administrators. The team engaged in pre-session briefings and after session debriefing to share challenges and lessons learnt on an on-going basis. The mode of operation and organization evolved with time. For a start, 4 beds with two workers per bed was arranged, two runner (bay) nurses to support them and one floor supervisor coordinator. In addition a specialist general surgeon provided the technical specialist support.

The runner nurses provided logistical support as was required e.g. giving extra supplies. The floor supervisor (coordinator) ensured proper identification of clients, flow of clients in and out of operating room and proper documentation. The surgeon supervised the ongoing procedures from bed to bed to ensure compliance to infection control standards among others. This was done through answering questions, coaching and appraising and ensuring efficiency and safety. Eventually this need of a roving surgeon was diminished as the team gained in confidence and proficiency.

Proficiency outside the operating room included appropriate and practical client schedules. The scheduled days for SMC were separate from the screening, counseling and registration days. The days for screening, counseling and registration were Tuesday and Thursday afternoon (2:00 to 6:00pm).The choice circumcision days were Friday and Saturday (7:00am to 6:00pm). The rationale for the weekend was to minimize disruption of the normal work routine of service delivery at the hospital as well as to ensure convenience for the clients.

## Methods

### Setting

The exercise was carried out at International Hospital Kampala (IHK), a 200 bed private hospital where 12 operating beds were dedicated to SMC activities. IHK is located in an urban setting, close to the central business district with a catchment of greater Kampala (with approximately 2.5million people).

### Participants

Participants were volunteers responding to a general call for free SMC. The call was implemented through word of mouth, short message service (SMS) and occasional radio announcements. The main advertising method was word of mouth. The circumcised men were encouraged to inform others to come. They were well briefed on the vital information to relate to others, radio announcements were made, flyers and pull up banners were also used. Posters were taken to a few secondary schools. Eligible participants were taken through a mandatory counseling process and offered the minimum package of safe male circumcision. The minimum package included: Voluntary Counseling & Testing (VCT), STI screening, STI treatment if indicated, condom supplies, and SMC general information and consent for circumcision under local anesthesia. Only males equal to or above 15 years were eligible. Those with co-morbidities including sickle cell disease, on treatment with chemotherapy, bleeding disorders and severe penile anomalies like hypospadias were excluded. Although most of the participants came from Makindye division, other divisions of Kampala were also represented.

### Materials

The main materials used included absorbable sutures, wound dressings, local anesthetic drugs, and oral analgesic drugs. These materials were secured from the Hospital Central Pharmacy through internal requisition orders. The Central Pharmacy procures materials using the already existing mechanisms and platform for procurement.

### Surgical Techniques

The team was composed of personnel who had prior surgical work experience. They had all worked in the operating room environment before; therefore they were well grounded in matters of infection control, aseptic technique, sluicing, waste segregation and disposal as well as safe handling of surgical instruments. This team was trained in stages, concentrating on technical skills through skills lab and hands on training including but not limited to knot tying, administration of local anesthetics and haemostasis. Apart from the technical skills, the theory, justification of SMC as well as attitudinal aspects of patient care were handled during the class room didactic sessions. The attitudinal aspects included dignity, respect and communication with the patients. During the training, three surgical techniques for circumcision were discussed; sleeve resection, dorsal slit and the forceps guided method. The sleeve method was the method of choice for the team because of what was deemed as superior cosmetic outcome and less bleeding.

The sleeve resection method was divided into several steps to ease instruction and learning. These included mapping over the corona with a surgical blade by performing a superficial cut. This superficial cut was achieved by using a surgical marker or inking with an hypodermic needle. The inner side (mucosal side) was “mapped” in a similar manner a residual cuff approximately 0.5-1cm was left behind.

Excising (referred to as trimming) the prepuce along the marked line followed the mapping. After trimming the standard four quadrant mattress stitches were placed. In addition a figure of eight at the frenulum and a varied number of in between simple sutures (8-12). A75cm long vicryl rapide 4/0 was used and this length of suture was mostly sufficient. In the absence of vicryl rapide, catgut 4/0 on a cutting needle was a substitute.

After placing the last stitch a dressing with ordinary dry sterile gauze rolled into a 3cm wide strip was placed over the suture line, and left on for 48-72 hours.

### Data Collection

Operating room logs, hotline logs and a structured questionnaire were used to collect data. In order to time this entire process of the SMC procedure, a stop clock was used to determine the amount of time taken to complete a circumcision. Timing started from applying the antiseptic solution to the surgical site and ended after the wound dressing was applied. A 48 hour personnel cover was available for adverse events management. Three hotlines with easy to memorize numbers were availed to all clients. Within 24 hours a dedicated phone operator/nurse called each one of the circumcised men to establish their state of well being using a standardized questionnaire. All patients returning with AEs, were classified according to a pre agreed format and managed according to protocol.

**Ethical considerations:** All patients were counseled and consented for the procedure. The 15 -17 years old had their parents or guardians consent on their behalf.

## Results

A total of 3000 men were circumcised in 27 days spread over a 4 months period (April to August 2011). Mean age was 25 years (SD 6.6), range 15 - 58 years, median 24 years, Circumcision procedures were carried out over the weekends with an average of 111 circumcisions per working day. The study recorded a 2.1% (mild) adverse events rate. No deaths occurred (Table 1, Table 2).

**Table 1:** Safe male circumcision work rate and adverse events using surgical circumcision at International Hospital Kampala, 20SMC12

Day	Beds	SMCsdone	SMCper bed	MeanOperating time	AE (with 24h)
1	4	37	9.3	55	3
2	8	61	7.6	50	8
3	9	102	11.3	36	3
4	10	133	13.3	30	1
5	9	123	13.7	45	2
6	5	44	8.8	42	1
7	7	88	12.5	-	0
8	5	66	13.2	37	1
9	7	81	11.6	36	0
10	9	132	14.7	-	2
11	7	100	14.3	33.5	7
12	5	82	16.4	35	3
13	6	100	16.7	36	1
14	10	20	2	38	1
15	12	187	15.6	36.9	4
16	6	136	22.7	35	3
17	5	91	18.2	36	2
18	8	159	19.9	31	2
19	7	127	18.1	34	7
20	8	125	15.6	31	2
21	10	139	13.9	33.5	3
22	8	125	15.6	32	2
23	7	124	17.7	34	2
24	8	128	16	29.8	1
25	8	152	19	30	0
26	8	130	16.4	-	0
27	11	183	16.6	29	0
**Totals**		**3000**	**14**	**32**	**52**

Mean operating time 32 min, output per bed was14 and Adverse events (AE) rate 2.1%Procedures took place between April – July 2011

**Table 2:** Safe male circumcision adverse events, STIs and other anomalies at International Hospital Kampala, Kampala, 2012

	N	(%)
**Complications**		
Bleeding (Oozing)	12	23
Haematomas	35	67
Sepsis (Infections)	5	10
**Anomalies**		
Xeroderma balanitis	3	0.1
Hypospadia (Grade I – II)	6	0.2
Phimosis	5	0.16
Peyronie's disease	2	0.01
**STIs**		
Penile Warts	5	0.16
Urethral discharges	6	0.2

### Mobilization

Out of the 3800 that registered, 3000 (79%) turned up. Of the 800 (21%) that did not turn up 100 (12.5%) were randomly sampled and interviewed by phone to establish reasons for not. They were asked 5 questions. The commonest reason for not turning up was failure to secure time off work or school (50%). Another 20% had rescheduled to a later date but had not returned and a few confessed to a change of mind.

### Adverse Events (AE)

The adverse events were all mild and reversible, they were mostly hematomas and active bleeding. The majority (47/52) occurred within the first 24 hours and reported in the evening of the procedure. Even though five infections were recorded, these were judged, on visual inspection, as such due to the presence of a pus discharge from the suture line; there was no gram staining or culture and sensitivity done to confirm the presence of active infection. Other complaints but not adverse events were multiple erections - reported as unusually uncomfortable and frequent within the first week. This was captured on the hotline conversations.

## Discussion

In this case study, a coalition between a private hospital and public organization formed a dual implementation partnership (PPP) that successfully carried out SMC at a rate of 111 per day with an adverse events rate of 2.1% at a single urban site in the heart of the city of Kampala. This AE rate is comparable to experiences elsewhere [12]. This PPP model is one of the ways resources may be distributed in the country in order to realize the enormous task of mass SMC that lies ahead of Uganda and the 12 other sub Saharan African countries. This approach may be scaled up in a number of ways by increasing sites with a different geographical and location by increasing the output at this urban site. Embracing Public Private Partnerships and collaboration (PPP & PPC) in the Health sector is important in light of the challenges the public sector faces in health care, finance, management and provision.

Turning to the private sector can, when appropriately structured and executed, help address specific cost and investigative challenges, deliver improvements in efficiency (e.g. improved service provision and management at reduced costs) and enhance service quality (e.g. increased expertise, more rapid and substantial investments in infrastructure, a potential to attract and retail better performing staff [13].

Leveraging partnerships to address public sector challenges may not be easy; these may take long to establish and make functional. In this case study of IDI/IHK, the establishment took four months of back and forth discussions but after that the implementation was immediate. The establishment was based on a contract that outlined common objectives, risks and rewards. The Private provider in this case was responsible for all the project operations at its site. It was a service contract for a defined service quality and efficiency was catered for through output and adverse events monitoring.

The perceived benefits of PPPs include: reduced spending, greater efficiency, leveraging expertise, performance based monitoring and incentives, technology transfer and reduced/better allocation of risk. IDI did not need to train IHK staff, IHK trained them, though technical support by way of providing reporting formats and data collection tools was provided. PPP arrangements are not without risks; the perceived risks included: creation of excess capacity and new capacity in the wrong place. There is indeed a risk of excess capacity in case uptake reduces or contracts terminate. However, with SMC this is unlikely to happen soon given the large national targets existing. In addition this trained workforce at IHK is employable wherever they may wish to go. IHK a leading health care provider had a proven track record of service delivery of high quality and had the public visibility and acceptance.

For implementers the choice of which private provider to chose may depend on the specific need and context. Financial stability of the potential partner, proven track record, expertise in the field and monitoring and evaluation capabilities are factors that should be considered. Shared values of work ethic, mutual trust and integrity also play a part.

### Operating time and preferred technique

**Figure 1:**
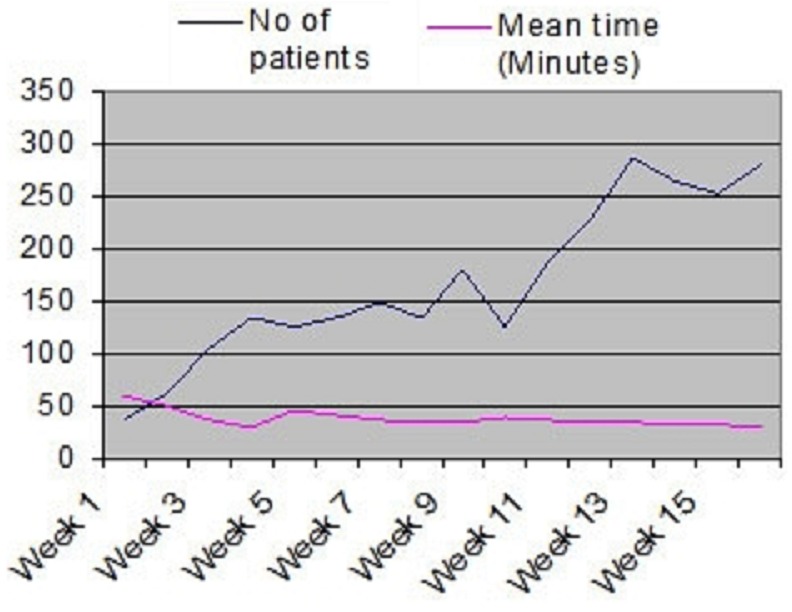
Number of patients and mean operating time for safe male circumcision at International Hospital Kampala, Kampala, 2012

Even though the overall mean operating time was 32 minutes while using the more technically challenging sleeve resection method and using ligatures for achieving haemostasis. With more practice (experience), the time reduced to less than 30 minutes as demonstrated in Figure 1. Apart from a few individuals nearly all operators were doing circumcision for the first time. Introduction of diathermy cauterization may further reduce this operating time which may be critical for improving work rate, though this needs to be scientifically investigated. The average work rate was 14 clients SMCs per bed. However this started as 7 clients and rose up to 23 over the weeks. A consistent 15 - 18 SMCs per table was realized in the last half of the period. This level of output was realized with more practice. The operating time dropped over the course of time from an average of 55 minutes to 29 minutes.

The fastest sleeve resection operator did so in 14 minutes. Most operators rejected the forceps guided method for two reasons: first there was more bleeding and hence taking up more time for meticulous haemostasis (and more use of suture materials, two pieces of vircyl rapide would be used in this situation - at a cost of $ 5 a piece) and secondly the forceps guided method seemed to leave behind excess mucosal cuff, which looked less cosmetically pleasing.

We are not sure what the impact of excess mucosal cuff could be in terms of HIV transmission after circumcision nor do we know how much “cuff” is safe to leave behind. It is reasonable to hypothesize that the more cuff left behind, the more HIV target cells are left and therefore more risk of HIV transmission [14, 15].

### Adverse events and penile anomalies

With all methods of male circumcision, we expected adverse events to occur. What is important is that these were few mild and completely reversible with minimal or no intervention. We recorded a 2.1% adverse events rate which is comparable to experiences else where. All events were mild and reversible, the majority occurring within 24 - 48 of the operation.

A hotline was available to all clients. Those that came back to the facility found a nurse waiting for them. Some only required a change of dressing, others release of stitches and others haemostasis. A number called back to complain of multiple and frequent uncomfortable erections; this could be due to initial hypersensitivity of the “naked” glans. This complaint faded away with the passage of time (4-6 weeks). Balaritis Xerotica Obliterans (BXO) [16] is a chronic sclerotic dermatitis involving the genital skin of men. Clinically patients with balanitis xerotica obliterans develop discrete, angular, white atrophic macules and patches on the glans, prepuce and foreskin of the penis with only rare involvement of the shaft. The prepuce is often thickened with fissures, erosions appear over the glans. Phimosis is a known sequel and there were two patients. BXO causes are unknown; however autoimmune and genetic factors are implicated. Even though BXO is confirmed by histologic evaluation in these cases the clinical picture was classic. Xeroderma Balanitis rate was 0.2% higher than that recorded in the literature [16, 17]. Tight phimosis with failure to retract the foreskin was found in 0.5%. There was no incidence of un-descended testis. Penile Peyronie's disease is a tissue disorder which affects 1-4% of men [18], in this case study, 2 patients were identified who had curved penises with visible scars at the mid shaft with no history of trauma.

### Participants, VCT and HIV prevalence

Participants were young men mostly in their early twenties. The HIV prevalence was 0.2%, much lower than the 7% for Kampala residents. This could have been because of the age bracket, young single men and mostly in school, though there could be other reasons that may need investigating. This presents as a window of opportunity for HIV prevention among this age group cohort in the communities they came from.

Mobilization for mass SMC is a crucial ingredient for success. Mobilization was done through word of mouth, SMS, radio and Facebook. The majority of clients who turned up indicated that they were encouraged by a friend who had already undergone SMC. A satisfied client brought in the next client. And therefore the need to focus on the SMC client's experience was important. Improving the client's experience may be achieved by making local anaesthesia (LA) infiltrations painless by using a small gauge needle (G25-6), by providing sufficient information and handling clients with courtesy and dignity during and after the procedure. In addition a hotline was available 24/7; (overnight cover) to attend to any on site post operative review by a physician was possible if and when needed.

### Lessons and gaps

It was possible to task shift the entire circumcision process to a pair of non physician cadre mostly clinical officers and graduate nurses. However this was preceded by a 6 week long training course spread over several weekends. Supervision, mentoring and coaching were sustained during the follow up period. The more challenging freehand sleeve resection method was mostly used with an average operating time of 32 minutes (without diathermy). Task shifting is possible and it worked. The general sense of employing non-physicians was one of empowerment and a great sense of job satisfaction. The appreciation of the responsibility of being in charge of an entire procedure of SMC was welcome.

The demand for SMC was prevalent in this urban setting site. In order to fulfill the demands for scale up aggressive mobilization needs to be done. This involves consideration of the convenience of the clients for example the time of week when SMC services are available for this urban population.

Scale up will impact and will require more operators, therefore more training. The absolute number of AE will increase implying more resources for AE management. More counselors shall be needed. The scale up will mean a need to procure large quantities of supplies for use as well as prepare the sites to accommodate large numbers of men e.g. (toilet facilities).

Central and bulk procurement for all consumables by implementation partners (IPs) is important to reduce cost and ensure availability of goods. Goods for use e.g. reusable kits may be centrally sterilized and distributed according to need. Exploration and adoption of surgical devices such as Prepex or Shang ring are recommended.

## Conclusion

Mass SMC male circumcision was done safely efficiently and cost effectively, through task shifting and a private public partnership.
